# Association between parental divorce and mental health outcomes among Lebanese adolescents: results of a national study

**DOI:** 10.1186/s12887-021-02926-3

**Published:** 2021-10-18

**Authors:** Sahar Obeid, Gloria Al Karaki, Chadia Haddad, Hala Sacre, Michel Soufia, Rabih Hallit, Pascale Salameh, Souheil Hallit

**Affiliations:** 1 Faculty of Arts and Sciences, Holy Spirit University of Kaslik (USEK), Jounieh, Lebanon; 2 INSPECT-LB: National Institute of Public Health, Clinical Epidemiology, and Toxicology, Beirut, Lebanon; 3 Faculty of Medicine and Medical Sciences, Holy Spirit University of Kaslik (USEK), Jounieh, Lebanon; 4 Research Department, Psychiatric Hospital of the Cross, Jal Eddib, Lebanon; 5 Université de Limoges, UMR 1094, Neuroépidémiologie Tropicale, Institut d’Epidémiologie et de Neurologie Tropicale, GEIST, 87000 Limoges, France; 6 University of Nicosia Medical School, Nicosia, Cyprus; 7 Faculty of Pharmacy, Lebanese University, Hadat, Lebanon

**Keywords:** Depression, Anxiety, Suicidal ideation, Divorce, Adolescents

## Abstract

**Background:**

In Lebanon, divorce rates have jumped from nearly 7000 in recent years to 8580 in 2017, an increase of 22.5%, with North Lebanon recording the highest number, followed by Beirut, likely resulting in increased behavioral problems in the offspring of divorced parents. Furthermore, one out of two Lebanese adolescents whose biological parents were divorced, separated, or deceased has a psychiatric disorder. More information regarding the impact of divorce on the mental health of Lebanese adolescents is still missing. The objective of this study was to explore the association between divorce and mental health outcomes, particularly depression, anxiety, and suicidal ideation among Lebanese adolescents.

**Methods:**

A cross-sectional study conducted between January and May 2019 enrolled 1810 adolescents aged 14 to 17 years, using a simple randomization method to choose schools. A proportionate number of schools was selected from each of the five Lebanese Mohafazat (Beirut, Mount Lebanon, North, South, and Beqaa), based on the list of the Ministry of Education and Higher Education. A total of 18 private schools were approached; two declined, and 16 accepted to participate.

**Results:**

The mean age of participants was 15.42 ± 1.14 years, with 53.3% females. After adjustment for the covariates (age, sex, and house crowding index), the results showed that adolescents whose parents are separated compared to living together had more social fear (Standardized Beta (SB = 0.270) and avoidance (SB = 0.188), higher depression (SB = 0.045), and higher suicidal ideation (SB = 0.370).

**Conclusion:**

Our findings reveal that teens with divorced parents had higher social fear and avoidance, depression, and suicidal ideation, highlighting the need for adequate prevention programs to support both children and parents during this emotionally difficult period.

## Background

Parental separation is a circumstance where both children and parents experience discomfort and pain, leading to intense stress. Thus, many children and adolescents may find it difficult to cope with their parents’ divorce. In Germany, in 2017, a considerable number of children went through a parental divorce [1]. According to the Center for Disease Control and Prevention (CDC), divorce rates in the United States had reached 787,251 in 2017, in 45 reporting States and D.C., with a divorce rate of 2.9 per 1000 population [2]. Divorce prevalence has also increased in European countries, with 2.6 per 1000 population per year in Denmark [3], 4.8 in Russia [4], and 2.1 in Belgium [3]. In Lebanon, divorce rates are 1.6 per 1000 population [4], and they have jumped from nearly 7000 in recent years to 8580 in 2017, an increase of 22.5%, with North Lebanon recording the highest number, followed by Beirut [5], likely resulting in increased behavioral problems in the offspring of divorced parents.

Divorce is the result of a decoupling process that begins even before a parent leaves the house and has severe repercussions in the future [6, 7]. Parents and children might be, thus, often confronted with specific stressors at the various stages of the divorce process even after it has occurred. Post-divorce stressors can include reduced financial resources, parental wrangling over custody, housing and neighborhood relocation, new school, and new friends. As for pre-divorce stressors, they considerably generate feelings of alienation and stacked dissatisfaction with the marital relationship [6]. On the road to divorce, these feelings can manifest and develop into devastating hostile or brutal parental interactions, painful for parents [8], but also with consequences on the well-being of children [9]. Broken and dysfunctional family relationships and the dreadful out-turn of divorce disruption could affect the mental health of children [10].

Many parents caught up by the burden of their divorce and struggling with their own emotions tend to become oblivious to their children’s need for support, whether emotional or physical, neglecting them because of lacking resources and time. Sometimes children are stuck between their parents; they experience difficult psychological situations and become drained from emotional abuse (“*If you love me, then you cannot love your father’s new girlfriend!*”). In this context, those children may try to reduce conflicts between their parents and feel triangulated between them, serving as messengers between both [11]. Additionally, parents can go through spikes of debilitating emotional stress resulting in severe mental health problems, consequently neglecting their children, which may impact their physical needs such as food, clothing, and health matters, including vaccination follow-up [12]. Furthermore, the intense emotional stress of the parents could become uncontrollable to the point where a child can be physically punished because of parental depression and irritability [13].

Interparental Conflict (IPC) following divorce consists of several factors, including anger, unsettled grief, hostile contempt, uncooperative co-parenting, verbal and physical fighting, and legal conflict between the parents [14, 15]. In the context of IPC, previous authors displayed the best outcomes in children with two high-quality parents and low IPC. Hence, young adults whose fathers had moderate contact/moderate support and low IPC had significantly higher academic achievement and marginally lower externalizing problems [16]. Nevertheless, some findings suggest that if granted adequate parenting time, quality parenting in one parent may be protective and lead to positive child outcomes even in the presence of IPC [16].

Studies have shown that the offspring of separated parents could involve in risky sexual behavior, live in poverty, and have adaptation problems reported as academic failures and difficulties (e.g., lower grades and quitting school), disruptive behavior (e.g., behavioral and drug addiction issues), depressed mood [17], miserable humor, and major anxiety disorder [10].

Several studies reported the link between the age of children at the onset of parental separation and the way they will adapt and cope with another model of a family [18, 19]. A child of 2 years old will not understand divorce, while a teenager can grasp the complexity of the parental decision to separate. Children and adolescents can experience various affective disorders and psychological repercussions, depending on their age, gender, maturity, and the presence of a support system. Indeed, teens with divorced parents may be more anxious [20, 21], depressed [17, 21], aggressive, stressed, delinquent, prone to addiction, and have more suicidal ideation [21]. Additionally, a nationally representative study of Norwegian 8-year-olds found that children of divorced parents are significantly overweight [22]. Moreover, children of separated parents have more emotional and behavioral problems and reduced academic and social performances [23].

Although parental divorce is a considerable public health problem for children and adolescents, it could be a positive change, especially when the child experiences a high conflict level. However, most children with divorced parents are resilient with no behavioral evidence of psychological problems. It is essential to recognize that those often experience worries and stress on occasions where both parents have to meet, such as graduation or marriage [24].

To the best of our knowledge, few studies showed the impact of divorce on adolescents in Lebanon. Maalouf et al. [25] found that one out of two adolescents whose biological parents were divorced, separated, or deceased has a psychiatric disorder, noting that these participants were only recruited from one district (Beirut). Hence, the need for a national representative study. This same study revealed that the 30-day prevalence of psychiatric disorders was 26.1%, with anxiety disorders (13.1%) and ADHD (10.2%) being the most prevalent disorders. Only 6% of those with disorders reported seeking professional help. Parental marital status, not attending school, having a chronic medical condition, having a family history of psychiatric disorders, and propensity to bullying and being victimized by peers emerged as correlates of having psychiatric disorders [25]. More information regarding the impact of divorce on the mental health of Lebanese adolescents is still missing. Therefore, the objective of this study was to explore the association between divorce and mental health outcomes, particularly depression, anxiety, and suicidal ideation among Lebanese adolescents. The practical implication of our study relates to evaluating the possible influence of previous or recent parental divorce on adolescent mental health to warn the parents about the importance of secure attachment.

## Methods

### Participants

A cross-sectional study conducted between January and May 2019 enrolled 1810 (90.5%) adolescents aged 14 to 17 years out of 2000 approached, using a proportionate random sampling of schools from all five Lebanese governorates (Beirut, Mount Lebanon, North, South, and Beqaa) based on the list of the Ministry of Education and Higher Education. A total of 18 private schools were approached; two declined, and 16 accepted to participate, distributed as follows: 4 in Beirut, 2 in South Lebanon, 6 in Mount Lebanon, 2 in North Lebanon, and 2 in Beqaa. The decision to include participants in this age interval only was due to accessibility and because this age represents a highly sensitive period in terms prevalence of mental disorders (e.g., depression, which affects 14.3% of youth aged 13 to 17 years) [26].

All students between 14 and 17 years old were eligible. They had the right to accept or refuse to enroll in the study; those who agreed to participate received no financial rewards in return. The methodology used in this study is the same as the one used in previous papers [27–32].

### Minimal sample size calculation

The Epi info software calculated a minimum sample size of 318 participants to have adequate power for the bivariate and multivariable analyses, based on 44.45 and 28.74% of anxiety disorder in adolescents with and without parental divorce, respectively [33].

### Questionnaire

The questionnaire used was anonymous and in Arabic, the native language in Lebanon; it required approximately 60 min to complete. Students filled it at school to eliminate parents influencing interventions. Completed questionnaires were collected back and sent for data entry.

The questionnaire consisted of two parts. The first one assessed sociodemographic characteristics of the participants, self-reported height and weight, to calculate the Body Mass Index (BMI), and the number of persons in the household and the number of rooms in the house, excluding the bathroom and the kitchen, to calculate the household crowding index (the number of rooms divided by the number of persons) [34].

The second part included the following scales:

#### The Liebowitz Social Anxiety Scale (LSAS)

The self-reported version used [35] is divided into two subscales (fear and avoidance) and includes 24 items graded on a Likert scale from 0 to 3 [36]. Higher scores indicate high social phobia. Scores below 30 indicate the absence of social anxiety disorder (SAD), whereas scores between 30 and 59 and 60 or more indicate the presence of probable and very probable SAD, respectively. In this study, Cronbach’s alpha values were excellent for the total score (0.969) and fear and avoidance subscales (0.952 and 0.951, respectively).

#### The Adolescent Depression Rating Scale (ADRS)

This 10-item scale was developed to screen for depression among adolescents, with questions rated as yes/no. Higher scores indicate higher levels of depression [37]. The cut-off value corresponding to the presence of depression according to the DSM-4 was 4 [37]. The Cronbach’s alpha value in this study was 0.940.

#### The Columbia-Suicide Severity Rating Scale (C-SSRS)

This 6-item tool, recently validated in Lebanon [38, 39], is used to assess suicidal ideation and behavior. A score of 0 indicates the absence of suicidal ideation, while a score of 1 or more confirms the opposite [40]. The Cronbach’s alpha value in this study was 0.966.

### Forward and back-translation

Two translators performed a forward and backward translation for all non-validated scales. One translated the scales from English into Arabic and the other one back into English. The two English versions were then compared to check for discrepancies, which were resolved by consensus.

### Statistical analysis

Data analysis was performed on SPSS software version 23. Cronbach’s alpha values were recorded for all the scales to assess internal consistency. A descriptive analysis was done using the counts and percentages for categorical variables and means and standard deviations for continuous measures. Missing values constituted less than 10% of the total data and thus were not replaced. Student t-test was used to compare two means, whereas the Pearson correlation test was used to correlate two continuous variables.

Taking each scale as a dependent variable, a multivariate analysis of covariance (MANCOVA) was carried out to compare multiple measures between the two groups of parents (living together vs. separate), considering potential confounding variables: age, sex, and house crowding index. A *p*-value of less than 0.05 was considered significant.

## Results

Of a total of 2000 questionnaires distributed, 1810 (81.0%) were completed and collected back. Table 1 summarizes the sociodemographic characteristics of the participants. The mean age was 15.42 ± 1.14 years, with 53.3% of females. Additionally, 11.9% of adolescents had separated/divorced parents. The results showed that 57.1% of the participants had depression; 43.4 and 2.4% had probable and very probable social fear, respectively. Furthermore, 52.3 and 3.7% had probable and very probable social avoidance, respectively. Finally, 28.9% of the participants had suicidal ideation.

**Table 1:** Sociodemographic characteristics of the sample population

	**Frequency (%)**
**Sex**	
Male	844 (46.7%)
Female	963 (53.3%)
**Parents status**	
Living together	1581(88.1%)
Separate	213 (11.9%)
**Smoking status**	
Yes	468 (25.9%)
No	1342 (74.1%)
	**Mean ± SD**
**Age (years)**	15.42 ± 1.14
**Body Mass Index (kg/m2)**	21.95 ± 4.21
**Household crowding index (**Persons / room)	1.01 ± 0.64

The description of the social avoidance and fear, depression, and suicidal ideation scores is summarized in Table 2.

**Table 2:** Description of the fear, avoidance, depression and suicidal ideation scores

	Fear	Avoidance	Depression	Suicidal ideation
Mean	26.18	30.95	4.65	1.02
Median	27	33	4.68	0
Standard deviation	16.15	16.94	2.10	1.84
Minimum	0	0	0	0
Maximum	72	72	10	5

No significant association was found between Leibowitz’s social fear scale and depression (r = 0.035; *p* = 0.162); however, a positive correlation was found between Leibowitz’s social avoidance scale and depression (*r* = 0.122; *p* < 0.001).

### Bivariate analysis

Having separated parents and being a smoker were associated with higher fear, avoidance, depression, and suicidal ideation in adolescents. Males had higher avoidance and depression, while females had higher suicidal ideation. Older age was significantly but weakly associated with less depression and suicidal ideation. In addition, a higher household crowding index was significantly but weakly associated with less fear and avoidance (Table 3).

**Table 3:** Bivariate analysis taking the Liebowitz (Fear and avoidance) scores, depression and suicidal ideation scores as the dependent variables

	**Liebowitz – fear score**	**Liebowitz – avoidance score**	**Depression**	**Suicidal ideation score**
	**Mean ± SD**	**Mean ± SD**	**Mean ± SD**	**Mean ± SD**
**Sex**				
Male	25.92 ± 15.88	32.10 ± 16.26	4.79 ± 2.10	0.77 ± 1.61
Female	26.39 ± 16.38	29.97 ± 17.45	4.52 ± 2.09	1.22 ± 1.99
*p-value*	0.563	**0.012**	**0.010**	**< 0.001**
**Parents status**				
Living together	24.52 ± 15.03	29.77 ± 16.35	4.69 ± 2.07	0.75 ± 1.57
Separated	37.73 ± 18.87	38.72 ± 18.76	4.35 ± 2.18	2.86 ± 2.41
*p-value*	**< 0.001**	**< 0.001**	**0.030**	**< 0.001**
**Smoking status**				
Yes	35.42 ± 15.81	38.59 ± 16.45	5.18 ± 2.69	2.84 ± 2.34
No	23.47 ± 15.23	28.62 ± 16.40	4.49 ± 1.87	0.51 ± 1.28
*p-value*	**< 0.001**	**< 0.001**	**< 0.001**	**< 0.001**
	**Correlation coefficient**	**Correlation coefficient**	**Correlation coefficient**	**Correlation coefficient**
**Age (years)**	*r* = 0.024	*r* = −0.032	*r* = − 0.154	*r* = − 0.068
*p-value*	0.340	0.206	**< 0.001**	**0.006**
**Household crowding index**	*r* = −0.134	*r* = − 0.117	*r* = 0.024	*r* = − 0.047
*p-value*	**< 0.001**	**< 0.001**	0.329	0.061

Numbers in bold indicate significant *p*-values; *r* Pearson correlation coefficient

**Fig. 1:**
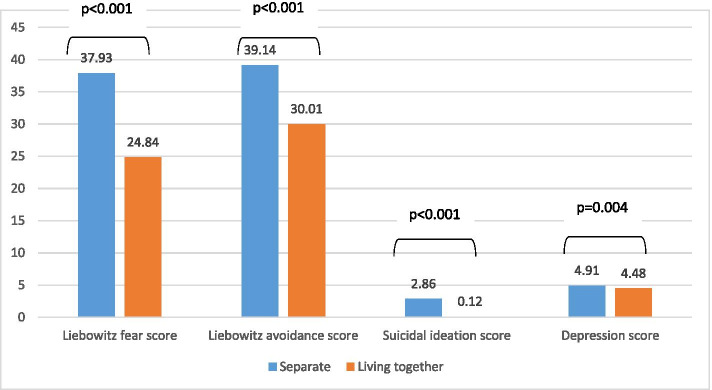
Mean values of the psychological scales according to the parents’ status, after adjustment for age, sex, and house crowding index

Figure 1 shows the mean values of all psychological scales according to the parents’ status after adjustment for age, sex, and house crowding index. The results showed that adolescents whose parents are separated had a significantly higher mean Leibowitz fear, avoidance, depression, and suicidal ideation scores, compared to those whose parents are living together.

### Multivariate analysis

The MANCOVA analysis was performed, taking the social avoidance and fear, depression, and suicidal ideation scales as the dependent variables and the parents’ status (separated vs. living together) as an independent variable; covariates included age, sex, and house crowding index. Adolescents whose parents are separated had more social fear (Standardized Beta (SB) = 0.270) and avoidance (SB = 0.188), higher depression (SB = 0.045), and higher suicidal ideation (SB = 0.370) compared to those whose parents are living together (Table 4).

**Table 4:** Multivariate analysis of covariance (MANCOVA)

	Unstandardized Beta	Standardized Beta	***p***-value	95% Confidence Interval	
				Lower Bound	Upper Bound
**Liebowitz – fear score**					
Age	0.611	0.043	0.066	−0.041	1.263
Sex (females vs. males^a^)	−0.656	−0.020	0.398	−2.180	0.867
Parents status (separated vs living together^a^)	13.190	0.270	< 0.001	10.899	15.482
House crowding index	−3.078	−0.127	< 0.001	−4.212	−1.944
**Liebowitz – avoidance score**					
Age	−0.216	−0.015	0.550	−0.927	0.494
Sex (females vs. males^a^)	−2.914	−0.086	0.001	−4.588	−1.240
Parents status (separated vs living together^a^)	9.472	0.188	< 0.001	7.000	11.943
House crowding index	−2.552	−0.102	< 0.001	−3.772	−1.332
**Total depression**					
Age	−0.295	−0.163	< 0.001	− 0.380	− 0.209
Sex (females vs. males^a^)	− 0.229	− 0.054	0.025	− 0.429	−0.028
Parents status (separated vs living together^a^)	−0.286	−0.045	0.004	−0.589	0.016
House crowding index	0.140	0.044	0.068	−0.010	0.291
**Suicidal ideation score**					
Age	−0.095	−0.060	0.009	−0.166	− 0.023
Sex (females vs. males^a^)	0.246	0.066	0.004	0.076	0.415
Parents status (separated vs living together^a^)	2.045	0.370	< 0.001	1.793	2.297
House crowding index	−0.085	−0.031	0.182	−0.210	0.040

In the global model, the independent variable is parents’ status (separated vs living together). Covariates are: age, sex, house crowding index

^a^Reference group

## Discussion

Divorce continues to be a problematic social experience in the Arab world, particularly in traditional communities, which may resent change and perceive any conflict as a threat to their integrity and social solidarity [41]. The practical implication of our study relates to evaluating the possible influence of parental divorce on adolescents’ mental health to warn the parents about the importance of secure attachment and quality parenting time. Thus, updating data on parental divorce would reduce the gap in the national literature. Furthermore, exploring the correlation between divorce and psychological distress among Lebanese adolescents is crucial to develop effective intervention programs.

This national study, targeting a group of schoolchildren from different regions representing the whole country, explored the link between parental divorce and its deleterious effect on mental health in adolescents. After adjusting for covariates, the results showed the association between divorce and social fear and avoidance, higher depression, and suicidal ideation.

The results of our study showed that 57.1% of the participants had depression; this rate is higher than the one obtained in a previous study conducted among a sample of Lebanese adolescents – 26% revealed severe depression [40]. Likewise, in our study, 43.4 and 2.4% had probable and very probable social fear, respectively. Furthermore, 52.3 and 3.7% had probable and very probable social avoidance, respectively. Finally, 28.9% of the participants had suicidal ideation, higher than the 16% obtained in 2005 on a sample of 5038 Lebanese adolescents. The latter study had addressed risk factors for suicidal ideations [42], i.e., poor mental health, alcohol use and drug abuse, victimization, and lack of parental understanding or support.

Previous studies revealed that 39.9% of adolescents with non-separated parents and 40.4% of those with separated parents suffered from long-term depression [43]. As for suicidal ideation, adult daughters of divorced parents had 83% higher odds of suicidal ideation than their female peers who had not experienced parental divorce [44]. Furthermore, the results of a study conducted in Spain (Galicia) showed that parental separation led to a mean increase of approximately 20% in depressive symptoms, anxiety (generalized), hostility, paranoid ideation, and interpersonal alienation [45]. Interparental conflicts, economic strain, family moves, and parental depression might explain the association between parental separation and adolescents’ mental health [43].

Our study emphasized the blatant correlation between parental divorce and social fear/avoidance, consistent with other studies explaining this correlation by the fact that the period preceding the divorce is most often marked by communication problems between parents, creating an atmosphere of pressure in the family and a feeling of unpredictability and tension for the child [20, 46]. These feelings have a significant association with the behavior of adolescents, who may disclose fears and anxieties and uncontrollable outraging bursts, especially in situations within the family. Children and adolescents may show intense specific fears in case this situation persists long enough. Parents sometimes respond to these fears by indifference or sarcasm, causing intimidated young people to conceal their fears and distress or even pretend not to have, which can result in social phobias or panic attacks in adolescence [47]. Moreover, as soon as the child discovers parental separation, the process of mourning begins. Fears for the future emerge, alongside confusion over which parent is less responsible for the divorce and with which parent the child is supposed to live. Such situations predispose the child to higher stress, which is a mediating factor for the development of fear and anxiety [48].

Our results also showed that parental divorce was significantly associated with higher depression, in line with those of other studies [49, 50]. Living in a single-parent family often involves limited resources, whether financial or social (support and parental supervision), which in turn are likely to be linked to poorer mental health in the offspring, such as depression [43]. It also results in reduced time spent with one of the parents, which usually means less involvement [51]. Furthermore, previous research revealed that the absence of the father could affect the psychological adjustment of boys more than girls. Boys are likely to need a male role model during adolescence. Also, initiating and maintaining close relations is more difficult for boys [50].

Parental divorce was associated with increased suicidal ideation in Lebanese adolescents, in line with previous findings [52–54]. Indeed, women who used to live with their fathers were significantly more likely to report lifetime suicide attempts than those who lived with their mothers [55]. Moreover, a previous study revealed that children staying with one of their parents did not experience more suicidal ideation compared to their peers [56]. However, children living without parents had higher stress and higher risk for mental disorders (suicidal ideation) than those living with a single parent [56]. Research indicates that suicidal risk in children of divorced parents is related to several factors, including ineffective parent-child communication, weak family cohesion, and insecure attachments [57].

### Clinical implications

Given the increased likelihood of disruption after parental divorce, the need for preventive measures is of primary importance. The mental health of adolescents with divorced parents should be cared for extensively by teachers and caretakers. These adolescents might benefit from qualified treatment and long follow-up periods. In addition to standard treatment like antidepressant medication and cognitive behavior therapy, other treatment/supportive strategies might be added, for instance, family interventions and, if needed, cooperation with the social services. For example, previous studies have shown that parental sensitivity can be strengthened and work as a buffer against the risk of future depressive episodes among children [58]. Another example, the New Beginnings Program (NBP), group-based intervention for divorced parents and their children, focuses on changing aspects of the child’s environment that directly involve the child, including increasing effective discipline strategies, improving parent-child relationship quality, and decreasing exposure to interparental conflict [59]. This program is not available in Lebanon; efforts should aim at implementing it in the country.

Furthermore, psychologists and health managers at schools should adopt closer monitoring and tailored approaches to provide a “substitute attachment figure” that could undoubtedly decrease the likelihood of anxiety and depression. For adolescents experiencing suicidal ideation, low feelings of belonging may represent an important treatment target in psychotherapy. Interventions focused on increasing belonging by supporting family interactions, strengthening positive peer relationships, or fostering relationships with mentors or other adults may be beneficial for decreasing adolescent suicide risk.

### Limitations

Due to the cross-sectional design of the study, assumptions about causality remain hypothetical. Information bias may be present since the data is based on self-reported answers. Moreover, some of the used scales are not validated in Lebanon to date. Selection bias is possible due to school selection, as public schools were not included in this study. The refusal rate can also predispose to attrition bias. A social desirability bias may also be present since adolescents may have answered in a way that seems socially acceptable. The use of a questionnaire can lead to information bias due to possible problems in understanding the questions and overestimation/underestimation of symptoms that may lead to inaccuracy. Additionally, a residual confounding bias is also likely since not all factors related to mental health among Lebanese adolescents have been taken into consideration in our study (e.g., conflict, pre-morbid diagnosis, genetic predisposition, among others). Nonetheless, the authors believe that the study results are meaningful, original on the national level, generalizable to the whole population, and consistent with the literature.

## Conclusion

Our findings reveal that teens with divorced parents had higher social fear and avoidance, depression, and suicidal ideation, highlighting the need for adequate prevention programs to support both children and parents during this emotionally difficult period. Consequently, enrolling separated parents in special prevention programs can guide them in creating a protective and supportive environment for their children. Further studies should focus on improving these programs to boost adolescents’ confidence and build a better society in the long run.

## Data Availability

The authors do not have the right to share any data information as per their institutions policies.

## Abbreviations

CDC: Center for Disease Control and Prevention
BMI: Body Mass Index
LSAS: Liebowitz Social Anxiety Scale
ADRS: Adolescent Depression Rating Scale
C-SSRS: Columbia–Suicide Severity Rating Scale
